# A Review on Diabulimia: Exploring the Intersection of Disordered Eating, Eating Disorders, Insulin Dose Manipulation, and Type 1 Diabetes

**DOI:** 10.2174/0115733998347278250309180707

**Published:** 2025-07-14

**Authors:** Velimir Altabas, Jelena Marinković Radošević, Nika Grubiješić

**Affiliations:** 1 Department of Endocrinology, Diabetes and Metabolic Diseases, Sestre Milosrdnice University Hospital Center, Zagreb, Croatia;; 2 School of Medicine, Zagreb, Croatia

**Keywords:** Diabulimia, type 1 diabetes, blood glucose monitoring, eating disorders, pancreatic β-cells, gestational diabetes mellitus (GDM)

## Abstract

**Introduction:**

Although insulin is essential for managing type 1 diabetes and is life-saving for patients with this condition, some individuals may intentionally reduce or omit insulin due to a fear of weight gain or a desire to lose weight. This behavior is commonly referred to as diabulimia.

**Methods:**

Since diabulimia is not formally defined, a systematic review of the limited literature was conducted on November 8th, 2024, using PubMed, Scopus, and Web of Science databases. The search terms included “diabulimia”, “insulin omission”, “insulin restriction”, “eating disorders”, “disordered eating”, and “type 1 diabetes”. Out of 288 manuscripts, 19 were selected after excluding non-English articles and screening the titles and abstracts.

**Results and Discussion:**

Eating disorders and disordered eating are common in patients with type 1 diabetes, often driven by concerns regarding body image and weight. These behaviors can complicate diabetes management, worsen glucose control, and increase the risk of complications. Diabulimia may develop as a coping mechanism, especially in adolescents with higher body mass index and a history of eating disorders. Diagnosis is challenging due to the lack of established guidelines, but poor glucose control can raise suspicion and prompt further psychological evaluation. A multidisciplinary approach, combining medical care, nutrition, mental health support, and therapy, is recommended, despite limited evidence.

**Conclusion:**

While diabulimia is not formally recognized, understanding its impact can help healthcare professionals diagnose and manage it more effectively, improving patients’ health and well-being.

## 1 INTRODUCTION

Diabetes mellitus is a group of long-term metabolic disorders marked by high blood glucose, resulting from a variety of causes and mechanisms and requiring tailored treatment plans. The most prevalent type is type 2 diabetes (T2DM), followed by type 1 diabetes (T1DM) and gestational diabetes mellitus (GDM). T2DM represents over 90% of cases worldwide, whereas GDM is confined to pregnancy. Although less common, T1DM affects over 9 million people globally and presents significant challenges due to its complex treatment needs and the risk of both acute and chronic complications. Type 1 diabetes arises from the autoimmune destruction of pancreatic β-cells, leading to insulin deficiency. Insulin therapy becomes crucial for patients with significant β-cell loss to maintain good glucose control, enabling them to preserve physical fitness, occupational functionality, and overall health [1-4]. Despite advancements in T1DM treatment, such as insulin analogs, automated insulin pumps, and continuous glucose monitoring systems, managing the condition remains challenging for patients due to the constant need for blood glucose monitoring and frequent dietary and insulin adjustments [5].

In more serious cases, constant glucose monitoring, dieting, carbohydrate counting, and insulin dose adjustments can lead to psychological problems, often referred to as diabetes distress. This term describes the emotional strain of living with diabetes and the frustration, burnout, and emotional burden of continual self-management. Diabetes stress can interfere with optimal glucose control, affect clinical outcomes, and further diminish the patient’s quality of life [6]. Additionally, imbalanced insulin therapy can lead to undesired side effects, such as hypoglycemia and weight gain [7].

Unwanted weight gain, diabetes distress, and patients' unmet perceptions of an acceptable physique are key factors contributing to the development of eating disorders and disordered eating behaviors in individuals with T1DM [8-10]. Eating disorders encompass a range of psychological conditions characterized by disturbances in eating behaviors and attitudes toward food and body image. Unrealistic expectations regarding physical beauty usually influence body image issues. Anorexia nervosa, bulimia nervosa, and binge-eating disorder are the most frequently diagnosed individual eating disorders [11].

While anorexia is characterized by self-starvation and binge eating disorder with episodes of excessive food consumption without a sense of control and possible weight gain, bulimia nervosa is associated with binges in food intake, followed by compensatory behaviors. These involve specific methods to restrict caloric utilization and keep body weight in balance after larger meals. Patients may induce vomiting, use laxatives, diuretics, or enemas (*e.g.*, purging type), or do excessive fasting and/or exercising (*e.g.*, non-purging type) [12]. The same techniques may also be used in patients suffering from the purging type of anorexia nervosa, but those patients typically have a low body mass index [13]. The symptoms and signs associated with the most common eating disorders are shown in Table **1**.

Eating disorders can have serious physical and psychological consequences, resulting in excess mortality [14]. The lifetime prevalence of eating disorders in the general population is estimated to be about 8.4% (3.3-18.6%) for women and 2.2% (0.8-6.5%) for men [15].

Disordered eating is a broader term related to eating disorders. It includes behaviors, such as emotional eating, night eating, and disinhibited eating, which may be associated with compensatory purging behaviors, but do not meet the criteria for a psychiatric diagnosis of an eating disorder [16].

The intersection between T1DM therapy and eating disorders gives rise to a specific condition known as diabulimia. Diabulimia refers to the deliberate manipulation of insulin doses by patients with diabetes to control or lose weight, often following episodes of excessive caloric intake [17]. At its core, diabulimia is a maladaptive coping mechanism observed in diabetic patients receiving insulin treatment who also have an eating disorder. This specific purging method involves intentionally omitting or reducing insulin doses to avoid its anabolic effects. The lack of insulin results in hyperglycemia, glycosuria, increased fat breakdown, dehydration, and subsequent weight loss [18-21]. As a result, glucose control worsens, reducing the likelihood of meeting the criteria for well-controlled diabetes in non-pregnant patients, as shown in Tables **2** and **3**.

Diabulimia has serious health risks since patients with T1DM are insulin-dependent, and decreasing or omitting insulin may result in the acute onset of life-threatening ketoacidosis, and long-term chronic microvascular and macrovascular complications, including premature death [23-27]. Other systemic health issues, like frequent infections and delayed wound healing, may also occur [28, 29].

Despite these possible deleterious consequences, diabulimia is still not recognized as a specific condition, within the context of eating disorders or disordered eating behaviors, even though diabetologists have been addressing eating disorders for decades [30]. Therefore, it is not surprising that, because diabulimia is widely underrecognized and understudied, specific guidelines are lacking [13, 29]. This review aimed to provide a comprehensive scientific overview of diabulimia, exploring its prevalence, underlying factors, clinical manifestations, health consequences, and available treatment options.

## 2 MATERIALS AND METHODS

A literature search was conducted on November 8, 2024, using the Medline National Library of Medicine, Web of Science, and Scopus databases. The search terms included 'diabulimia', 'insulin restriction', 'insulin omission', 'eating disorders', 'disordered eating', and 'type 1 diabetes mellitus', used in various combinations. The specific search combinations were as follows:

Diabulimia AND Eating disorders AND Type 1 diabetes mellitus

Diabulimia AND Disordered eating AND Type 1 diabetes mellitus

Insulin restriction AND Eating disorders AND Type 1 diabetes mellitus

Insulin restriction AND Disordered eating AND Type 1 diabetes mellitus

Insulin omission AND Eating disorders AND Type 1 diabetes mellitus

Insulin omission AND Disordered eating AND Type 1 diabetes mellitus

This search identified a total of 288 manuscripts. After duplicates were removed, 103 articles remained for further screening. Following an initial review of titles and abstracts, 5 non-English papers and 10 book chapters were excluded. Full-text articles were then retrieved and independently assessed for inclusion in the review by the three reviewers (V.A., N.G., and J.M.R.). Studies were included if they used a tool to screen for or identify disordered eating behaviors, eating disorders, diabulimia, or insulin restriction and/or omission in patients with T1D, and reported specific outcomes related to the tool.

Research studies that focused on other types of diabetes, as well as reviews, notes, editorials, letters, and case reports, were excluded. Studies were also excluded if they lacked a screening or assessment tool or did not report tool-related outcomes.

Finally, a total of 19 manuscripts were selected for inclusion, and the main meta-data from these articles are presented in the relevant sections of this review.

## 3 RESULTS AND DISCUSSION

### 3.1 Prevalence and Risk Factors of Diabulimia

The general opinion is that eating disorders and disordered eating are more frequent in patients with T1DM and are related to significant health risks [20, 31, 32]. A well-conducted earlier meta-analysis reported a 3-fold increase of bulimia nervosa in patients with T1D compared to controls [30].

However, evidence regarding eating disorders and disordered eating in T1DM does not uniformly support this statement. Some research studies claim fewer disordered eating behaviors and less body dissatisfaction in adolescents with T1DM compared to healthy peers. Indeed, a large cohort of poorly controlled diabetic adolescents refused to participate in one of the studies [33, 34]. Another study, using a less precise methodology, also reported that diabetic youth with long-standing diabetes (over 10 years) do not suffer more often from disturbed eating behaviors [35]. In contrast, another research work indicated that diabulimia is relatively common among T1DM patients across various cultures and continents [36-42]. Studies reporting diabulimia prevalence are shown in Table **4**.

As shown, prevalence estimates vary, with one study reporting rates of insulin misuse in over 65% of patients with T1DM suffering from eating disorders [49]. It should be noted that these estimates can differ based on the study population, diagnostic criteria, and methodology [18]. These findings should be cautiously extrapolated to a broader T1DM population, as this represents a key limitation of existing studies. Also, the severity of disturbed eating behaviors may vary over time in the same subjects, as reported in a prospective study [58].

Diverse risk factors have been associated with diabulimia. While not every individual with T1DM suffers from diabulimia, certain factors may increase susceptibility to this disorder. These risk factors include increasing body mass index (BMI), body image dissatisfaction, younger age, previous history or familial history of eating disorders, mental disorders, diabetes distress, poor diabetes control, and social and peer pressure [8-10, 58, 59].

An increase in BMI from adolescence to adulthood has been linked to heightened concerns regarding shape and weight, as well as greater dietary restraint, particularly among female patients with T1DM. While overt eating disorders were not more prevalent in these patients compared to the general population, milder forms of disordered eating were common and had implications for diabetes management [48]. It is not clear if the female gender may be an additional risk factor since a serious bias appears in recognizing eating disorders in males [18, 60]. One study found no gender differences in diabulimia prevalence in patients with T1DM [18], despite the generally accepted fact that female gender is a risk factor for eating disorders [48, 61, 15]. Table **5** presents research studies correlating gender and other risk factors with diabulimia prevalence.

Nevertheless, evidence shows that weight loss concerns and disordered eating behaviors are more extensively studied in the female population [46, 62-64].

Patients with T1DM who have body image dissatisfaction issues or concerns about weight and appearance may be at a higher risk of developing diabulimia [20, 65]. An observational follow-up study supported these findings, demonstrating that strong extrinsic appearance-focused motivation and lower self-efficacy are associated with more prevalent disturbed eating behaviors in adolescents with T1DM. In contrast, intrinsic motivation focused on health outcomes showed no association with disturbed eating behavior [66]. Social pressures, media influence, and internalized ideals of thinness can contribute to this risk [10]. In addition, certain personality factors, such as neurotic perfectionism and borderline traits, like dependent personality characteristics, are associated with disturbed eating behavior, insulin manipulation, and suboptimal glycemic control [64]. However, the associations with diabulimia remain unexplored.

Adolescence represents a critical period for the development of diabulimia. The challenges of transitioning into adulthood, body image concerns during adolescence, and the desire for independence in diabetes management can contribute to the risk [52]. Nevertheless, disturbed eating behaviors and insulin omission may also be seen even in pre-teens and in the older adult population with T1DM [9, 42, 67].

Patients with a history of other eating disorders, such as bulimia nervosa or binge-eating disorder, are at an increased risk of disordered eating behavior. If an eating disorder precedes the onset of diabetes, diabetes management can be more complicated and diabulimia may develop. In addition, if a first-degree family member suffers from eating disorders, the risk of developing diabulimia is increased [9, 68]. Certain preexisting mental issues, like anxiety and depression, are associated with the development of disturbed eating behavior in patients with T1DM [69, 70]. In addition, children with T1DM and their parents frequently experience high levels of depression and anxiety [71, 72]. Therefore, T1DM could be regarded as a family disease, with recent studies indicating that parental anxiety, depression, and distress are linked to depression in their children and poor glycemic control [71]. The correlation between these potential risk factors and diabulimia still needs to be studied.

In addition, diabetes distress and specific difficulties in managing blood glucose, including frequent hypoglycemia, and anxiety about insulin-related weight gain can contribute to the development of diabulimia as individuals seek to regain a sense of control over their bodies and overall diabetes management [39, 59, 73]. Peer pressure associated with stigmatization, social comparisons, and the desire to fit in with peers who also may have eating behavior disorders or body image concerns may influence the development of diabulimia, but these interconnections require further exploration [10, 74, 75]. It is important to point out that individuals may experience a combination of conditions that increase their vulnerability. Identifying these risk factors can help healthcare professionals be vigilant in detecting diabulimia and providing appropriate support and intervention.

### 3.2 Detection and Diagnosis of Diabulimia

Early detection and diagnosis of diabulimia are essential for timely intervention and comprehensive treatment. Healthcare professionals should address concerns related to disordered eating and diabetes management during the routine assessment of patients with T1DM, especially for patients with poor diabetes control [32].

Unfortunately, the signs and symptoms of diabulimia overlap with those of both T1DM and some eating disorders, like binge-eating disorder or bulimia nervosa. Patients with diabulimia frequently engage in secretive behaviors related to insulin manipulation, making it even more challenging to detect and diagnose the condition. Shame, guilt, fear of judgment, and concerns about losing control over diabetes management can contribute to the delay in diagnosis [32, 76]. Common warning signs and symptoms indicating the presence of diabulimia are shown in Table **6**.

In general, excessive focus on weight and body shape and unusual weight fluctuations in patients with T1DM may indicate the need for further psychosocial assessment to evaluate the individual's psychological well-being, including body image concerns, self-esteem, mood disorders, and stress related to diabetes management [32, 76-81].

Chronically elevated HbA1c levels that are inconsistent with reported insulin use, along with positive urinary ketones or low time-in-range values from continuous glucose monitoring (CGM), should also raise suspicion in this context. However, the results related to CGM data require further confirmation [41, 57, 82]. Studies showing a correlation between elevated HbA1c and the presence of diabulimia are presented in Table **7**.

Further clinical evaluation should focus on disordered eating behaviors and attitudes, binge eating episodes, purging behaviors, and psychological distress related to eating and body image. Assessing for eating disorders, such as bulimia nervosa, is important as diabulimia can co-occur or evolve from a pre-existing eating disorder [68, 76].

The specific screening tool for eating disorders in the diabetic population is the Diabetes Eating Problems Survey-Revised (DEPS-R), developed in 2010. Although it has limitations related to time consumption and is unsuitable for routine clinical practice, this tool provided more specific information than generic screening instruments and identified more young people with eating disorders than clinician reports [76, 83, 84]. This questionnaire has been successfully validated over time in different patient groups from various cultures, but its score addresses eating disorders in general, not specifically diabulimia, as only one of the 16 questions pertains to skipping insulin related to meals [54, 67, 69, 84-88]. However, patients with T1DM who scored higher on this survey were found to have poorer glucose control parameters [54, 69, 89]. In addition, patients at high risk for diabetes-specific eating disorders demonstrated a significantly higher risk for other psychiatric conditions, such as anxiety and depression. Binary logistic regression analysis revealed that higher anxiety and depression scores could predict higher future DEPS-R scores [90]. A recent study showed that patients with T1DM and higher DEPS-R scores were more likely to be hospitalized for ketoacidosis in the past 6 months [38].

Another screening tool, the modified SCOFF questionnaire (mSCOFF) is more practical for routine clinical use. Nonetheless, this questionnaire does not specifically address the types of disturbed eating behaviors or insulin manipulation and lacks specificity for diabulimia [53, 91].

It is important to point out that psychological assessment is crucial in diagnosing eating disorders, but is insufficient for diagnosing diabulimia. While the mSCOFF questionnaire does not address diabulimia at all, the DEPS-R questionnaire addresses it only marginally. Additionally, there is more evidence linking worse diabetic control with the DEPS-R score compared to the mSCOFF. Therefore, despite its limitations, the DEPS-R questionnaire remains a better diagnostic tool. Another important limitation is that it lacks a specific focus on diabulimia, and its validity is heavily influenced by the overrepresentation of female adolescent samples, making it difficult to generalize the results to other populations [40].

While several studies have shown a correlation between eating disorders detected by these questionnaires and elevated HbA1c levels, one study analyzing CGM data found no factors related to glycemic control associated with likely eating disorders [54, 57, 69].

Importantly, the results obtained from questionnaires need to be interpreted within the broader clinical context. Therefore, developing new diagnostic tools that combine diabulimia-specific questions with laboratory results could enhance the recognition of this condition.

In short, diabulimia remains challenging to diagnose due to the lack of specific diagnostic criteria in medical or psychiatric guidelines, leaving healthcare professionals to rely on clinical judgment, along with medical and psychological assessments [17].

### 3.3 Treatment Approach

Early intervention is important for individuals suspected of having diabulimia [32]. The primary care physician plays a vital role in detecting early signs of diabulimia, coordinating overall care, monitoring general health, addressing comorbidities, and providing ongoing support [92].

Importantly, insulin restriction and poor glucose control do not necessarily indicate diabulimia; they may simply reflect non-compliance with insulin regimens for other reasons, so other potential causes must be excluded [53].

To the best of our knowledge, no large, multicenter randomized clinical trials addressing diabulimia have been published. However, expert opinions suggest that a multidisciplinary treatment model is essential for effectively addressing diabulimia. Therefore, a collaboration among healthcare providers specializing in diabetes management, mental health professionals, dietitians, and others in the treatment team is important to assess the diagnosis and comprehensive needs of individuals with diabulimia [81, 93].

Several components are typically included in a comprehensive multidisciplinary treatment approach, such as medical treatment, mental health support, and eating disorder management, including cognitive behavioral therapy, nutritional counseling, family therapy, and support groups. Ideally, healthcare specialists with primary expertise in diabetes should have some training in psychology, psychiatry, and eating disorders. Conversely, specialists in psychology and psychiatry could benefit from a deeper understanding of diabetes treatment [74, 93-95]. If obesity is a problem, anti-obesity drugs may be prescribed [96]. There is evidence that at least some of these drugs may alleviate binge eating disorder, though their impact on diabulimia remains unknown [97]. Registered dietitians with expertise in diabetes management and eating disorders can address specific concerns related to insulin manipulation and provide guidance on diabetes self-management, helping patients develop a healthy relationship with food and weight control [98].

The primary therapeutic focus is on mental health professionals experienced in treating eating disorders, who should address body image concerns, disordered eating thoughts and behaviors, depression, anxiety, and other psychological challenges. Cognitive behavioral therapy (CBT), specifically tailored for eating disorders, may be utilized to target erroneous thoughts and beliefs about body image, food, and insulin management, and develop appropriate coping strategies. Disappointingly, in a single-center randomized trial, psychoeducation strategies based on a standardized intervention proven effective for eating disorders showed no impact on insulin manipulation or diabetes control [99].

However, educating individuals and their families about eating disorders and disordered eating, body image issues, and mental health can help reduce stigma, increase awareness, and foster a more supportive environment [17, 28, 100]. Although several psychiatric drugs and medication classes, aside from anti-obesity drugs, have been used to alleviate eating disorders, no studies are available on their use for diabulimia [97, 101, 102].

Furthermore, engaging in support groups or peer networks specifically focused on diabetes and eating disorders can provide individuals with diabulimia a sense of community, validation, and shared experiences. Peer support can also offer practical tips, coping strategies, and encouragement throughout the recovery process [74].

### 3.4 Limitations of Available Evidence

Over the past four decades, numerous studies have been published on various aspects of diabulimia. However, the methodology, study populations, and study aims have shown significant heterogeneity.

Only a minority of studies have included a control group of peers without T1DM, specifically 4 out of the selected 19 studies included in this review [20, 35, 44, 52]. This presents a limitation in understanding potential differences in the prevalence of eating disorders between individuals with T1DM and those without diabetes. It is important to note that diabulimia is unique to individuals with diabetes who use insulin.

Most studies have employed a cross-sectional design, with only two follow-up studies, limiting the ability to track the progression of this condition over time, as presented in Table **4**. Additionally, various diagnostic tools have been used to identify eating disorders, reflecting changes in diagnostic and statistical manuals as well as advancements in diagnostic tools. Notably, since 2010, approximately half of the studies have used either the DEPS-R or the mSCOFF questionnaire, enhancing the comparability of findings in more recent research [35, 52-55, 57].

The target populations in these studies have been predominantly youth. Specifically, 6 out of 19 studies have exclusively involved diabetic youth aged 11-19 years [35, 45, 48, 50, 52, 54], 5 have included both adolescents and young adults up to 30 years of age [33, 44, 49, 53, 55], and 2 studies have focused on children up to 14 years of age [42, 43]. Consequently, individuals over 30 years of age have been underrepresented, with only a single study focusing on patients aged over 35 years [57].

Across all studies, reported HbA1c values have varied from 7% to 11.6% [45, 57]. Lower mean HbA1c levels have been observed in more recent studies, potentially reflecting stricter criteria for optimal diabetes control and advancements in treatment options over time. Importantly, since elevated HbA1c levels are linked to an increased risk of diabulimia, research focusing on populations with lower HbA1c values may inadvertently exclude patients suffering from this condition. In addition, different treatment strategies involving various generations of insulin have not been directly compared, limiting an understanding of their potential impact on diabulimia.

Interestingly, the mean BMI reported across studies has been generally below 25 kg/m^2^, except in two studies involving older adults. Older studies have tended to report lower mean BMI values compared to more recent ones, where a substantial proportion of participants have been classified as overweight or obese. Regarding weight reporting, some studies involving children and adolescents have reported weight or BMI percentiles [42, 54], while those focusing on older adolescents and adults have used BMI. Despite inconsistent BMI reporting, overweight and obese individuals with T1DM seem to be underrepresented, even though they face additional distress in managing blood glucose due to increased body weight [103-108]. Finally, underreporting has not been a primary outcome in any of the selected studies.

## CONCLUSION

Diabulimia is a relatively understudied condition with limited research, leaving several significant knowledge gaps in this field. Diabulimia studies are typically conducted in specialized treatment settings or specific populations, limiting the generalizability of findings to broader populations and contributing to the absence of proper treatment guidelines. Overall, female adolescents are overrepresented in the study populations. Studies with diverse populations are needed to understand better the prevalence, risk factors, outcomes, and treatment approaches for diabulimia. Follow-up studies tracking individuals with diabulimia over extended periods are scarce. Still, existing evidence indicates a higher rate of chronic microvascular complications, such as diabetic foot and chronic diabetic kidney disease.

Some of the key research gaps and limitations in diabulimia studies include a lack of consensus on diagnostic tools and criteria, self-report bias, limited research, and non-standardized treatment options. This lack of consensus hampers accurate diagnosis, making it challenging to compare findings across studies and impeding progress in understanding the condition. In addition, individuals may underreport or deny their behaviors due to shame, guilt, or fear of judgment. This self-report bias can impact the accuracy and reliability of the data collected.

Finally, the optimal treatment approaches for diabulimia are not well-established. Comparative studies evaluating various treatment modalities, such as psychotherapy, family therapy, and cognitive-behavioral interventions, combined with medical management, generally show no improvements. However, according to some initial research, new anti-obesity drugs, such as GLP-1 and GIP receptor agonists, may be used in the future to treat obesity in these patients and address body image concerns.

## Figures and Tables

**Table 1:** Characteristics, symptoms, and signs of commonly diagnosed eating disorders [12, 13].

**Eating Behavior**	**Anorexia Nervosa**	**Bulimia Nervosa**	**Binge Eating**
Main characteristics	Severely limiting calories or not consuming certain kinds of foods or food groups Intense fear of gaining weight despite low body mass Occasional use of purging methods and/or excessive exercise	Episodes of binge eating, commonly followed by using purging methods Feelings of guilt and/or shame leading to the use of various purging methods	Eating food in a short amount of time, without a sense of control when not hungry, and no purging Feelings of guilt and shame resulting in hiding while eating; feeling disgusted with oneself
Body mass	Low body mass index	Not characteristic	Increased body mass developing over time
Associated symptoms	Menstrual cycle irregularities in females Dehydration resulting in low blood pressure Hair and nail abnormalities Cold intolerance Low muscle mass Oesophageal reflux (in patients who vomit) Constipation Bloating and fullness after meals Osteopenia or osteoporosis Stress fractures related to excessive exercising Poor concentration Fatigue Irritability Depression and anxiety	Sore throat due to vomiting Dental decay resulting from erosion of tooth enamel by stomach acid Heartburn and gastroesophageal reflux Unexplained diarrhea Dehydration resulting in low blood pressure	-

**Table 2:** Criteria for good glycemic control in non-pregnant patients with diabetes [22].

**HbA1c**	**< 7.0% (53 mmol/mol)**
Preprandial capillary plasma glucose	4.4 - 7.2 mmol/L
Peak prandial capillary plasma glucose	< 10 mmol/L

**Table 3:** Criteria for good glycemic control in non-pregnant diabetic patients who use continuous glucose monitoring systems [22].

**Glucose management indicator (GMI)**	**< 7,0%**
Time in range (3.9 - 10 mmol/L)	>70%
Time below range (< 3.9 mmol/L)	< 4%
Time below (3.0 mmol/L)	< 1%

**Table 4:** Diabulimia prevalence reported in research studies.

**Authors**	**Study Design**	**Description of the Study Population**	**Diabulimia Prevalence**
Powers *et al.*, 1990 [43]	Cross-sectional	97 teenagers with T1DM	14% of girls 4.1% of boys
Fairburn *et al.*, 1991 [44]	Cross-sectional	100 adults with T1DM	37% of women 0% of men
Peveler *et al.*, 1992 [45]	Cross-sectional	76 adolescents with T1DM	15%
Polonsky *et al.*, 1994 [46]	Cross-sectional	341 female patients with T1DM aged 13 to 60 years	31%
Mannucci *et al.*, 1995 [47]	Cross-sectional	118 adult patients with T1DM	12.9%
Bryden *et al.*, 1999 [48]	8 years follow-up	76 adolescents with T1DM	30% of girls 0% of boys
Takii *et al.*, 2008 [49]	Cross-sectional	109 adult females with T1DM and eating disorders	67.9% reported severe insulin omission
Ackard *et al.*, 2008 [33]	Cross-sectional	143 adolescents with T1DM	1.4%/1.4% of boys for insulin restriction/omission 10.3%/ 7,4% of girls for insulin restriction/ omission
d’Emden *et al.*, 2013 [50]	Cross-sectional	124 adolescents with T1DM	5.6%
Philippi *et al.*, 2013 [51]	Cross-sectional	189 individuals with T1DM aged 12 to 59 years	8.5%
Wisting *et al.*, 2013 [52]	Cross-sectional	770 children and adolescents with T1DM aged 11 to 19 years	31.6% of individuals with insulin restriction 6.9% of individuals with insulin omission
Baechle *et al.*, 2014 [35]	Cross-sectional	629 children and adolescents with early-onset T1DM of at least 10 years duration	20.5% of girls 18.5% of boys
Baechle *et al.*, 2016 [53]	Cross-sectional	819 T1DM youth aged 11 to 21 years	7%
Falcao *et al.*, 2017 [20]	Cross-sectional	55 adults with T1DM aged 18 to 30 years	17.6%
Araia *et al.*, 2017 [54]	Cross-sectional	477 adolescents with T1DM	19%
Cherubini *et al.*, 2018 [55]	Cross-sectional	163 adolescents with T1DM	5.2%
Peducci *et al.*, 2018 [42]	Cross-sectional	85 children with T1DM aged 8 to 14 years	17.6%
Ip *et al.*, 2023 [56]	Cross-sectional	225 adults with T1DM	8.9%
Albaladejo *et al.*, 2023 [57]	Follow-up	198 adults with T1DM treated with insulin pumps	39%

**Table 5:** Studies on risk factors for diabulimia.

**Authors**	**Major Findings**
Powers *et al.*, 1990 [43]	Diabulimia is more common in girls.
Fairburn *et al.*, 1991 [44]	Diabulimia is detected exclusively in women.
Bryden *et al.*, 1999 [48]	Girls are more likely to omit insulin.
Ackard *et al.*, 2008 [33]	Girls are more likely to restrict and omit insulin. Girls are more likely to report body dissatisfaction.
Philippi *et al.*, 2013 [51]	Eating disorders increase the risk of insulin restriction/omission.
Wisting *et al.*, 2013 [52]	Adolescent age, increased body mass index, and disturbed eating behaviors increase the risk for diabulimia.
Baechle *et al.*, 2016 [35]	No significant gender-based differences exist in insulin misuse.
Falcao *et al.*, 2017 [20]	Increased body mass index, body dissatisfaction, and female gender are predictors of eating disorders.

**Table 6:** Common warning signs and symptoms indicating the presence of diabulimia.

Psychological symptoms	Body image issues Previous history of eating disorders Known psychiatric conditions Substance misuse Positive family history of eating disorders or psychiatric illness Diabetes distress Mood swings
Physical symptoms	Blurred vision Fatigue Body weight fluctuations Frequent urination Nocturia Frequent urinary infections Vomiting Dehydration Frequent abdominal pain Fruity body odor Slow healing of cuts and sores Frequent infections Symptoms due to other purging behaviors (*e.g.*, decayed teeth, heartburn, diarrhea)
Diabetes management	Poor diabetes control Poor glucose monitoring habits Less-than-expected insulin utilization Not showing up to scheduled appointments at the diabetes clinic Frequent hospitalizations for ketoacidosis or hyperglycemia Early occurrence of chronic diabetic complications
Social behavior	Decline in social and occupational functioning

**Table 7:** Research studies demonstrating interconnections between elevated HbA1c levels and diabulimia.

**Authors**	**Major Findings**
Mannucci *et al.* [47]	Higher HbAlc values were found in patients with clinical or subclinical eating disorders, including those with diabulimia.
Araia *et al.* [54]	Body mass index, HbA1c, insulin omission, and binge-eating frequency were moderately associated with higher DEPS-R scores in both genders.

## LIST OF ABBREVIATIONS

BMI: Body Mass Index
CGM: Continuous Glucose Monitoring
DEPS-R: Diabetes Eating Problems Survey-Revised
GDM: Gestational Diabetes Mellitus
GIP: Glucose-dependent Insulinotropic Polypeptide
GLP-1: Glucagon-like Peptide 1
GMI: Glucose Management Indicator
HbA1c: Glycated Hemoglobin
T1DM: Type 1 Diabetes Mellitus
T2DM: Type 2 Diabetes Mellitus
